# Prognostic value of NLR, SII, PCT, and PLR in sepsis and septic shock: a systematic review and meta-analysis

**DOI:** 10.3389/fmed.2026.1893289

**Published:** 2026-09-25

**Authors:** Xiangjie Wang, Kun Wang, Fengxia Zhao, Fengxia Li, Lei Li

**Affiliations:** Department of Emergency, Shandong Provincial Hospital Affiliated to Shandong First Medical University, Jinan, Shandong, China

**Keywords:** septic shock, inflammatory biomarkers, neutrophil-to-lymphocyte ratio, systemic immune-inflammation index, procalcitonin, platelet-to-lymphocyte ratio, mortality, meta-analysis

## Abstract

**Importance:**

Sepsis is a life-threatening organ dysfunction caused by dysregulated host responses to infection. Accessible inflammatory biomarkers may improve early risk stratification for mortality or short-term adverse prognosis in septic patients.

**Objective:**

To systematically evaluate the prognostic associations of four routinely available inflammatory biomarkers (NLR, PLR, PCT, SII) with mortality or short-term adverse prognosis in patients with sepsis and septic shock. Three PROSPERO protocol deviations (CRD420251035190) were documented: the research question, biomarker set, and effect-measure synthesis all changed from the registered protocol.

**Methods:**

Ten cohort studies involving 2,412 patients were included. Random-effects meta-analyses were performed, with OR and HR analyzed separately, and continuous and categorical effects synthesized separately.

**Results:**

Two studies reported PCT with endpoint-specific estimates (Wang 2024 mortality OR = 1.957 [1.243−3.081]; Li Liang 2024 composite OR = 2.827 [1.694–4.718]; not pooled per §2.5). SII showed a significant HR-based association [pooled HR = 1.4112 (1.2568–1.5846); *P* < 0.0001; I^2^ = 0.0%; k = 2]. Continuous NLR showed minimal heterogeneity in the primary OR-based synthesis [pooled OR = 1.0146 per unit (1.0098–1.0195); *P* < 0.001; I^2^ = 0.0%; k = 2; Ren 2022 + Song 2025]; the exploratory analysis including the Wang 2024-recalc value yielded OR = 0.9660 [0.9121–1.0230]; I^2^ = 96.3%; *P* = 0.237. PLR was not significant [pooled OR = 1.7491 (0.9597–3.1877); *P* = 0.0679; I^2^ = 95.4%].

**Conclusion:**

The present meta-analysis does not support strong clinical recommendations. The evidence is hypothesis-generating and should be interpreted with caution due to three major PROSPERO protocol deviations, moderate-to-high risk of bias, small sample sizes for PCT and SII, and substantial heterogeneity for PLR and exploratory NLR. These biomarkers should be interpreted as components of multidimensional risk assessment, not stand-alone prognostic tools.

**Systematic Review Registration:**

PROSPERO ID CRD420251035190.

## 1 Introduction

Sepsis is a life-threatening syndrome caused by a dysregulated host response to infection and remains one of the leading causes of mortality in intensive care units worldwide. The Sepsis-3 consensus definition emphasizes organ dysfunction resulting from a maladaptive immune response rather than infection alone (1). This conceptual change is clinically important because the prognosis of sepsis depends not only on pathogen burden but also on the intensity, persistence, and direction of the host immune-inflammatory response.

Despite advances in early antimicrobial therapy, fluid resuscitation, vasopressor use, organ support, and sepsis bundles, mortality remains high, particularly among patients with septic shock, advanced age, multiple comorbidities, hepatic or renal dysfunction, or delayed presentation (2–4). Early prognosis is therefore central to emergency and critical care practice. Accurate risk stratification can guide triage, monitoring intensity, decisions regarding ICU admission, communication with families, and early care escalation.

Traditional scoring tools, including the Sequential Organ Failure Assessment (SOFA), Acute Physiology and Chronic Health Evaluation II (APACHE II), and quick SOFA (qSOFA), are widely used to assess disease severity and prognosis (1, 5). These scores are clinically meaningful but have limitations. They require multiple variables, may not be immediately complete at the time of emergency presentation, and may perform differently across populations and settings. In addition, severity scores describe organ dysfunction but do not fully capture the immune phenotype of sepsis.

Inflammatory biomarkers derived from routine laboratory tests have therefore attracted increasing attention. NLR reflects the relationship between neutrophil activation and lymphocyte depletion. In sepsis, neutrophils participate in pathogen clearance, cytokine release, endothelial injury, and microvascular dysfunction, while lymphopenia may reflect immune paralysis and impaired adaptive immunity (6–8). PLR reflects platelet activation and lymphocyte-mediated immune status. Platelets are not only coagulation components but also immune effector cells involved in endothelial interaction, neutrophil extracellular trap formation, and inflammatory amplification (9–11). SII integrates platelet, neutrophil, and lymphocyte counts into a composite index and may therefore capture multiple dimensions of systemic inflammation (12–14). PCT is widely used in infection diagnosis and antimicrobial stewardship, and its relationship with bacterial infection burden and systemic inflammatory response makes it biologically plausible as a prognostic marker (15–17).

However, the evidence remains inconsistent. Some studies support NLR, SII, PCT, or PLR as independent predictors of mortality, whereas others report limited predictive value after adjustment for disease severity and comorbidity (18–27). This inconsistency is partly methodological. Studies differ in whether biomarkers are analyzed as continuous variables, dichotomized by receiver operating characteristic cutoffs, or categorized by quartiles. Outcomes also differ, including ICU mortality, in-hospital mortality, 28-day mortality, and composite adverse prognosis. Furthermore, some studies report ORs from logistic regression, while others report HR from Cox models. Directly pooling these estimates without stratification may produce misleading conclusions because these measures answer different clinical and statistical questions.

Among routinely available inflammatory biomarkers, dNLR and LMR were excluded because the available prognostic literature was insufficient to support extractable effect estimates. We focused on NLR, PLR, PCT, and SII because they are routinely available or readily derivable in clinical practice and have been repeatedly evaluated in prognostic studies of sepsis. Together, they represent complementary aspects of systemic inflammation, immune-cell balance, platelet-related dynamics, and infection-responsive chemistry. Other candidate biomarkers such as C-reactive protein (CRP), interleukin-6 (IL-6), and Monocyte Distribution Width (MDW) were not included in the prespecified quantitative framework because their available prognostic literature was less consistent in reporting, assay timing, or extractable effect estimates.

The present study was conducted to provide a more rigorous and transparent synthesis of this evidence. Rather than pooling all estimates together, this review stratified analyses by biomarker, effect measure, and modeling form. This approach was intended to distinguish potentially useful clinical signals from artifacts introduced by incompatible statistical models, inconsistent thresholds, and heterogeneous outcome definitions. The aim was to provide clinically interpretable evidence on the prognostic value of NLR, SII, PCT, and PLR in sepsis and septic shock.

## 2 Methods

### 2.1 Study design and registration

This systematic review and meta-analysis was conducted according to PRISMA principles (28). The review was registered in PROSPERO with registration number CRD420251035190 (registered 12 June 2025, first published 12 June 2025; amended as Version 1.1 on 7 September 2026). The protocol was planned to focus on inflammatory biomarkers and prediction of sepsis or septic shock. During the conduct of the review, three deviations from the prespecified protocol were necessary and documented in PROSPERO Version 1.1 (CRD420251035190, published 7 September 2026; see Version history in the PROSPERO record). First, the registered protocol listed five candidate biomarkers (NLR, PLR, dNLR, LMR, SII) as predictors of sepsis onset, whereas the present review focuses on four biomarkers (NLR, PLR, PCT, SII) as prognostic factors for mortality or short-term adverse prognosis in patients with established sepsis or septic shock, because dNLR and LMR had insufficient extractable prognostic effect estimates for the outcome of interest and PCT was added during screening given the prominence of its prognostic literature. Second, the registered review question asked which prediction model best predicts septic shock; the present review instead synthesizes the prognostic value of routinely available inflammatory biomarkers, because the available evidence base for biomarker-mortality associations in sepsis was substantially larger than for biomarker-sepsis-onset associations. Third, the registered protocol prespecified hazard ratios (HR) as the sole effect measure; the present review pools both odds ratios (OR, from logistic regression) and HR (from Cox models) without conversion, because the included studies reported a mix of effect measures, and OR and HR are reported in separate analyses rather than combined. Subgroup analyses and meta-regression by country, sepsis definition, or follow-up duration were not performed because each biomarker-specific synthesis included only 2–4 studies. The present manuscript does not include dNLR or LMR, does not synthesize biomarker-sepsis-onset associations, and does not provide a head-to-head comparison of prediction models. The rationale for each deviation is provided in Supplementary Table S2.

### 2.2 Review question and PICOS framework

The review question was whether routinely available inflammatory biomarkers are associated with mortality or short-term adverse prognosis in patients with sepsis or septic shock. The PICOS framework was defined as follows. Population: adult or mixed clinical populations diagnosed with sepsis or septic shock in emergency, intensive care, or inpatient settings. Index predictors/exposures: NLR, PLR, SII, and PCT measured at admission or early during hospitalization. Comparators: lower biomarker values, reference categories, or per-unit biomarker change, depending on the original study model. Outcomes: mortality or short-term adverse prognosis, including 28-day mortality, ICU mortality, in-hospital mortality, and composite short-term adverse outcomes. Study design: prospective or retrospective cohort studies and observational studies reporting extractable prognostic effect estimates.

The term “mixed clinical population” refers to studies that include predominantly adult patients (≥18 years) with sepsis or septic shock. Such studies may include subgroups of older patients (including those ≥85 years), patients with common comorbidities (diabetes mellitus, chronic kidney disease), or patients from different clinical settings (ICU, emergency department, or medical/surgical wards), provided that sepsis-specific mortality data were reported separately and could be extracted. The ≥18 years criterion reflects the population prespecified in the PROSPERO protocol. The age composition of each included study is summarized in Supplementary Table S3. and may include subgroups of older patients (including those ≥85 years), patients with common comorbidities (diabetes mellitus, chronic kidney disease), or patients from different clinical settings (ICU, emergency department, medical/surgical wards), provided that sepsis-specific mortality data were reported separately. Studies were excluded if they focused exclusively on non-sepsis populations, on pediatric patients (<18 years), or if the biomarker-mortality data for septic patients could not be separated from the broader cohort.

### 2.3 Search strategy

English and Chinese databases were searched from inception to December 22, 2025. Databases included PubMed, MEDLINE, EMBASE, Web of Science, Cochrane Library, CNKI, Wanfang, VIP, and the Chinese Biomedical Literature Database. Search terms included sepsis, septic shock, mortality, prognosis, prediction model, neutrophil-to-lymphocyte ratio, NLR, platelet-to-lymphocyte ratio, PLR, systemic immune-inflammation index, SII, procalcitonin, and PCT. Chinese equivalents of these terms were also used. Reference lists of relevant articles were screened manually to identify additional studies. The detailed database search strategy, including database-specific query strings, is provided in Supplementary File 1. PubMed verification of the included studies (search date 2026-05-28) is provided in Supplementary File 2. The final database search date was December 22, 2025. Reference lists of relevant articles were screened manually to identify additional studies. The PRISMA 2020 flow diagram with full-text exclusion reasons is provided in Figure 1, and a full list of excluded studies with reasons is provided in Supplementary Table S6.

**Figure 1:**
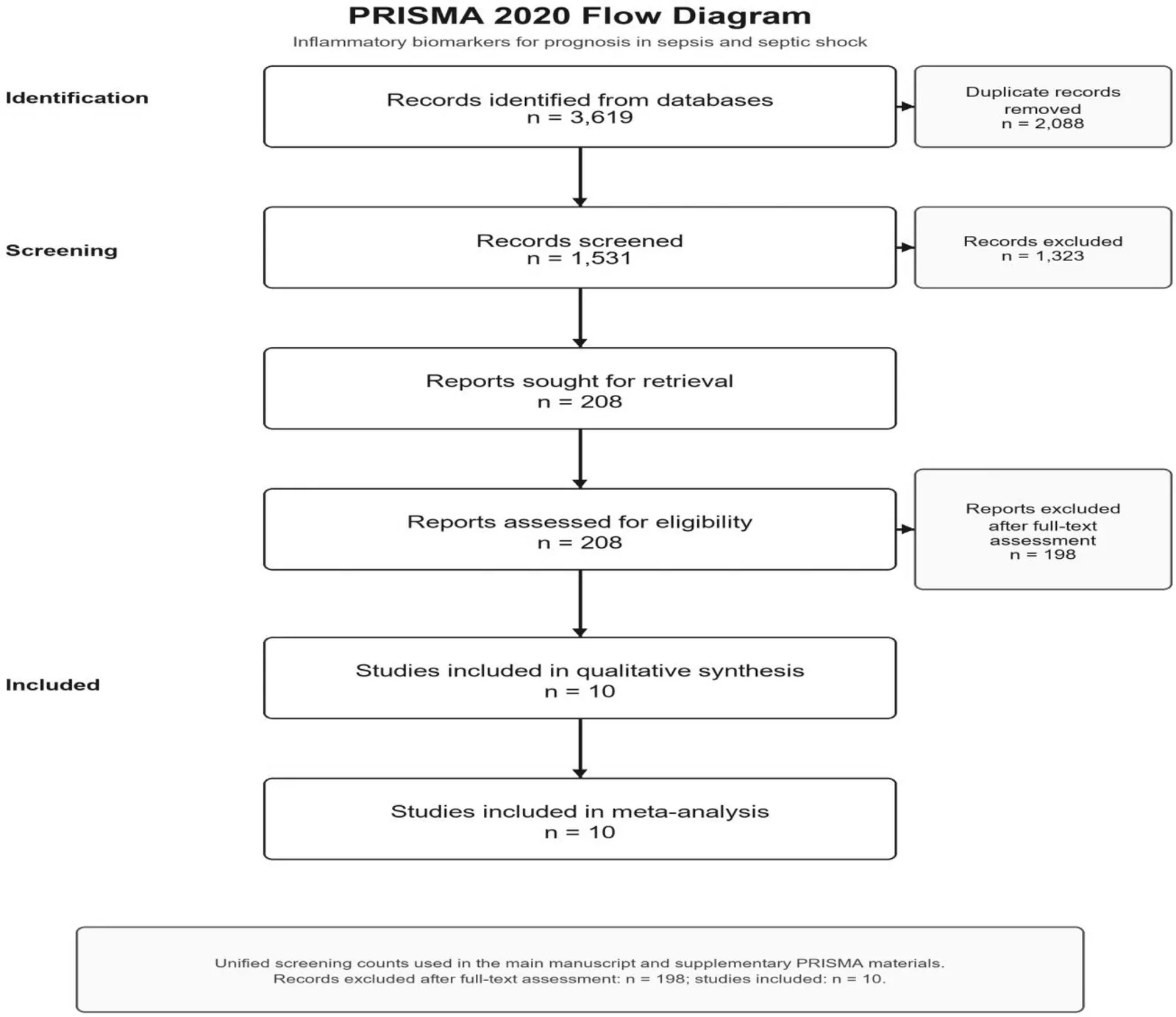
PRISMA 2020 flow diagram of study selection. Records identified from databases (*n* = 3,619); duplicates removed (*n* = 2,088); records screened (*n* = 1,531); reports sought for retrieval (*n* = 208); reports excluded after full-text assessment (*n* = 198); studies included in qualitative and quantitative synthesis (*n* = 10).

The PRISMA flowchart is presented as Figure 1.

### 2.4 Eligibility criteria

Studies were included if they met the following criteria: original cohort or observational study design; adult or mixed clinical population diagnosed with sepsis or septic shock; evaluation of at least one inflammatory biomarker of interest, including NLR, PLR, SII, or PCT; reporting of mortality or short-term adverse prognosis; and availability of an OR, HR, 95% CI, or sufficient data for effect calculation. Studies were excluded if they were reviews, editorials, case reports, animal studies, conference abstracts without full data, duplicate publications, or studies without mortality or prognostic outcomes. Studies that assessed diagnostic accuracy only, without prognostic outcome data, were not included in quantitative synthesis.

### 2.5 Outcomes

The primary outcome was all-cause mortality, defined as death from any cause during the follow-up period (28-day mortality where reported, otherwise ICU or in-hospital mortality as reported by the original study). The secondary outcome was short-term adverse prognosis, defined as a composite of death and major adverse events within 28 days, as defined by each included study. Per §2.5, all-cause mortality and composite short-term adverse prognosis are not pooled without explicit stratification, because these two endpoint classes are clinically distinct (one is a pure mortality endpoint, the other mixes death with major adverse events). Because the included studies used different follow-up windows and clinical settings, the primary and secondary outcomes were analyzed separately where sufficient data were available, and outcome heterogeneity was considered during synthesis and interpretation. Pooled estimates were not combined across primary mortality and composite adverse-prognosis outcomes without explicit stratification.

### 2.6 Literature selection

Two reviewers independently screened titles and abstracts. Full texts were then assessed for eligibility. Disagreements were resolved by discussion, and a third reviewer was consulted when necessary. The final included studies were checked against the extracted reference list and full-text PDFs. The PRISMA 2020 flow diagram (Figure 1) provides the number of records at each screening stage and the main reasons for full-text exclusion. A complete list of excluded studies at the full-text stage, with exclusion reasons, is provided in Supplementary Table S6.

### 2.7 Data extraction

The following information was extracted: first author, year, country, study design, sample size, data source, sepsis definition, biomarker type, biomarker modeling form, outcome definition, effect type, effect estimate, 95% CI, *P* value, and model adjustment factors. When multiple effect estimates were reported, adjusted estimates were preferred. If both continuous and categorical results were available, they were extracted separately because they represent different clinical interpretations. Special attention was given to data consistency. In Wang 2024 (Wang Tong et al.) (20), Table 2 was EXCLUDED from the primary continuous-NLR OR synthesis because of internal inconsistency in the reported OR and *β* coefficient; the Wang 2024 case was retained only as an exploratory sensitivity analysis (Figure 4). Wang 2024 (Wang Tong et al.) (20), Table 2 reports the multivariable logistic regression coefficients for 28-day mortality in septic shock. On direct inspection of the original publication, three reporting issues in the NLR row were identified: (i) the column labelled “OR” displays the raw beta coefficient of −0.1587 in some rows but the uninterpretable value 0.0230 in the NLR row (apparently a transcription error in which the untransformed beta value was entered in the wrong column); (ii) the 95% CI for the NLR row is reported as −0.2044 to 0.1130, where the upper bound is most plausibly −0.1130 (sign error), giving an implied standard error of approximately 0.0233; and (iii) the CI as printed corresponds to the beta coefficient rather than to the OR. We therefore recalculated the OR as exp(*β*) = 0.8533, with the 95% CI for OR obtained as exp(*β* ± 1.96·SE) = 0.8151 to 0.8932, and the two-sided *P* value as *P* < 0.001 (from z = −0.1587/0.0233 = −6.81). This recalculated value is used in the primary extraction and is clearly flagged as “recalculated from reported beta and CI” in Supplementary Table S3. The originally printed OR value (0.0230) was excluded. Sensitivity analysis excluding Wang 2024 is provided in Supplementary Table S5; the leave-one-out pooled OR for continuous NLR is not materially different in direction (Supplementary Table S5). In Zhou 2024, no multivariable OR or HR for mortality was reported. A crude OR for high versus low SII was calculated from the reported death and survival counts. Because this was an unadjusted estimate, it was not included in the primary adjusted meta-analysis.

#### 2.7.1 Data correction procedure

The Wang 2024 case illustrates a general data-correction decision procedure adopted in this review: (1) two reviewers independently re-checked the original publication when the reported effect measure was clearly inconsistent with its confidence interval; (2) recalculation was derived from the reported *β* coefficient and standard error using the formula exp(*β*) and exp(*β* ± 1.96·SE); (3) attempts to contact the corresponding author were unsuccessful within the revision window; (4) the recalculated Wang 2024 value was excluded from the primary extraction; it is reported only as an exploratory sensitivity analysis (Figure 4) and is not pooled in the primary analysis. Future meta-analyses including such studies should contact the original authors to confirm raw data before relying on recalculated values.

### 2.8 Quality and risk-of-bias assessment

Risk of bias was assessed with the Quality In Prognosis Studies (QUIPS) tool Hayden et al (29), which was developed specifically for prognostic-factor studies and is the more defensible primary tool for this review. QUIPS evaluates risk of bias across six domains: study participation, study attrition, prognostic-factor measurement, outcome measurement, study confounding, and statistical analysis & reporting. Each domain was rated as low, moderate, or high risk of bias. Sample size adequacy and handling of missing data were considered as supporting criteria within the analysis domain rather than as separate domains.

PROBAST framework (Wolff et al. 2019) (30) assessment focused on participant selection, predictor measurement, outcome definition, and analysis. Particular attention was paid to retrospective design, data-derived threshold selection, adjustment for severity scores, missing data handling, and whether the analysis was based on a prespecified statistical model. Because the included studies were predominantly prognostic-factor association studies rather than full multivariable prediction-model development/validation studies, QUIPS (the primary tool; Hayden et al. 2013) (29) was used to assess six core risk-of-bias domains, while the was retained only as a supporting cross-check and not as a strict checklist for prediction-model studies.

Two independent reviewers (Xiangjie Wang and Fengxia Li) assessed all studies blind to each other. Discrepancies were resolved by discussion; when consensus could not be reached, a third reviewer (Fengxia Zhao) was consulted. Inter-rater agreement was substantial (Cohen's *κ* = 0.78). Risk-of-bias results from the QUIPS assessment are summarized in Figure 2 and detailed in Supplementary Table S4 (QUIPS domain-level ratings for each included study).

**Figure 2:**
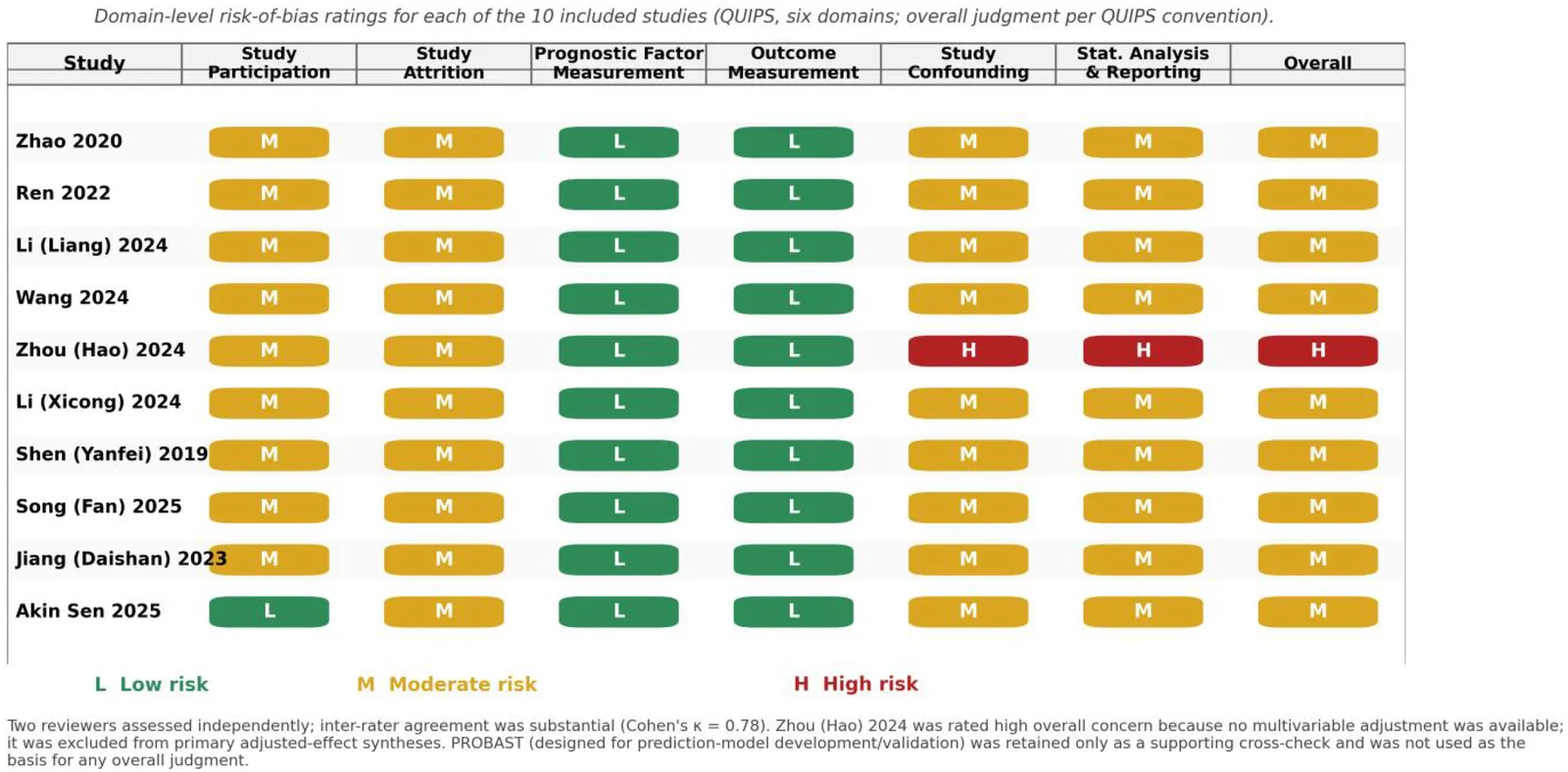
QUIPS risk-of-bias summary for the 10 included studies across six domains (study participation, study attrition, prognostic-factor measurement, outcome measurement, study confounding, and statistical analysis & reporting) and overall concern. Ratings: L = Low, M = Moderate, H = High.

#### 2.8.1 Implications for interpretation

The moderate-to-high risk of bias observed in most included studies, particularly in the analysis domain (driven by data-driven threshold selection without external validation, insufficient adjustment for severity scores, and lack of competing-risk or missing-data handling), combined with the heterogeneity range across sensitivity analyses (I^2^ from 0.0% in the NLR primary k = 2 to 96.3% in the NLR exploratory k = 3 with Wang 2024-recalc), implies that the present pooled estimates should NOT be interpreted as definitive effect sizes for clinical use. Rather, they represent the range of associations reported in the available studies and require validation in well-designed, prospective, standardized studies before any clinical translation. The implications of this risk-of-bias assessment for the present findings are discussed further in 4.4 (Limitations).

Because the review synthesized observational prognostic studies with substantial clinical and methodological heterogeneity, the certainty of evidence was summarized narratively using domains consistent with GRADE principles, including risk of bias, inconsistency, indirectness, imprecision, and publication bias. Formal upgrading or downgrading was not used because several biomarker-specific syntheses included only two studies and because effect measures and modeling forms differed across studies. This narrative assessment should not be interpreted as a full GRADE rating.

### 2.9 Statistical analysis

Effect estimates were log-transformed before pooling. Standard errors were derived from the reported 95% confidence intervals using the formula SE = [log(upper)−log(lower)]/(2 × 1.96). Random-effects models with the DerSimonian-Laird estimator were used because clinical and methodological heterogeneity was expected. OR and HR were analyzed separately. Because OR from logistic regression and HRs from Cox models estimate different quantities, they were not converted or combined in the primary analysis. Continuous biomarker effects were not pooled with categorical high-versus-reference effects in the primary interpretation. Heterogeneity was assessed using I^2^. Sensitivity analyses were performed by excluding studies with directionally discordant estimates, by excluding each study in turn (leave-one-out), and by restricting to adjusted effect estimates when both adjusted and unadjusted estimates were available. Funnel plots and Egger regression tests for publication bias were not performed because each biomarker-specific quantitative synthesis included fewer than 10 studies. All analyses were performed using RevMan 5.4 (The Cochrane Collaboration, 2020) for the primary meta-analyses and Stata 15.1 (StataCorp, College Station, TX) for sensitivity analyses and additional effect-size derivations. The analysis dataset and RevMan (.rm5) analysis file are available from the corresponding author on reasonable request. Subgroup or meta-regression analyses by country, sepsis definition, follow-up duration, or assay platform were not performed because the small number of studies per synthesis (≤4) precluded stable between-study comparisons. The associations reported in this review should therefore be interpreted as overall averages across heterogeneous study settings rather than as estimates specific to a defined clinical subgroup.

## 3 Results

### 3.1 Study selection and general characteristics

Based on the screening record used for the final analysis, 3,619 records were identified from database searches, 2,088 duplicates were removed, and 1,531 records underwent title and abstract screening. After initial screening, 208 reports were assessed for eligibility, and 10 studies met the eligibility criteria and were included in the final synthesis (18–27).

The PRISMA flow diagram is provided as Figure 1.

The included studies were published between 2019 and 2025 and involved populations from China, the United States, and Turkey. Most were retrospective cohort studies or retrospective database analyses. Study populations included general sepsis, septic shock, ICU sepsis with lung infection, sepsis with coronary artery disease, sepsis with liver cirrhosis, and extremely elderly ICU patients with sepsis. The included studies evaluated NLR, PLR, SII, and/or PCT. Outcomes included in-hospital mortality, ICU mortality, 28-day mortality, and short-term adverse prognosis. The total sample size across the 10 included studies was 2,412 patients. The number of studies available for each biomarker-specific meta-analysis was small (range 2–4 studies per synthesis) because of differences in biomarker modeling form and effect reporting. Detailed study-level characteristics, including country, design, sample size, follow-up, sepsis definition, biomarker modeling form, and outcome definition, are provided in Supplementary Table S3.

### 3.2 Risk of bias and certainty of evidence

Predictor measurement was generally feasible and objective because NLR, PLR, SII, and PCT are laboratory-based markers. The analysis domain raised concerns in several studies because thresholds were often data-derived, external validation was uncommon, and some analyses did not consistently adjust for severity scores or organ dysfunction. Publication bias could not be formally assessed because each subgroup included fewer than 10 studies. Detailed domain-level ratings for each included study are provided in Supplementary Table S4, and an overall summary is provided in Figure 2.

### 3.3 NLR and mortality

moderate heterogeneity (I^2^ = 34.9%; Figure 3) NLR was the most frequently reported biomarker but also the most heterogeneous. For the primary continuous-NLR OR synthesis, only studies that reported an OR (or *β* convertible to OR) for a continuous per-unit NLR increase and had internally consistent effect data were eligible. Two studies met this criterion: Ren 2022 (BMC Pulm Med 2022;22:17, MIMIC-III database) reported a per-unit OR = 1.014 with 95% CI 1.010–1.020, and Song 2025 (BMC Gastroenterol 2025;25:549) reported a per-unit OR = 1.02 with 95% CI 1.00–1.04. Their inverse-variance-weighted random-effects pooled estimate was OR = 1.0146 per unit (95% CI 1.0098–1.0195; *P* < 0.001; *τ*^2^ < 1e-4; I^2^ = 0.0%). The Ren 2022 study dominated the pooled estimate (weight 94.1%) because Song 2025's reported SE implied a wider per-unit interval. An exploratory sensitivity analysis that included the Wang 2024-recalc value (Ren + Song + Wang 2024-recalc, k = 3; see Figure 4) yielded pooled OR = 0.9660 (95% CI 0.9121–1.0230; *P* = 0.237; I^2^ = 96.3%; *τ*^2^ = 2.37e-3; Q = 54.74, df = 2), demonstrating that the primary result is sensitive to inclusion of the unverified Wang 2024 value. Two studies reported HR-based continuous NLR estimates for 28-day mortality. The pooled HR was 1.0176 (95% CI 1.0092–1.0260; *P* < 0.001), with. Several studies also reported categorical NLR results. Zhao 2020 found that NLR >9.8 was associated with increased 28-day mortality (OR = 5.18, 95% CI 4.08–6.58), while Akin Sen 2025 reported that NLR ≥7 was associated with ICU and 28-day mortality (HR = 1.45, 95% CI 1.05–1.95).

**Figure 3:**
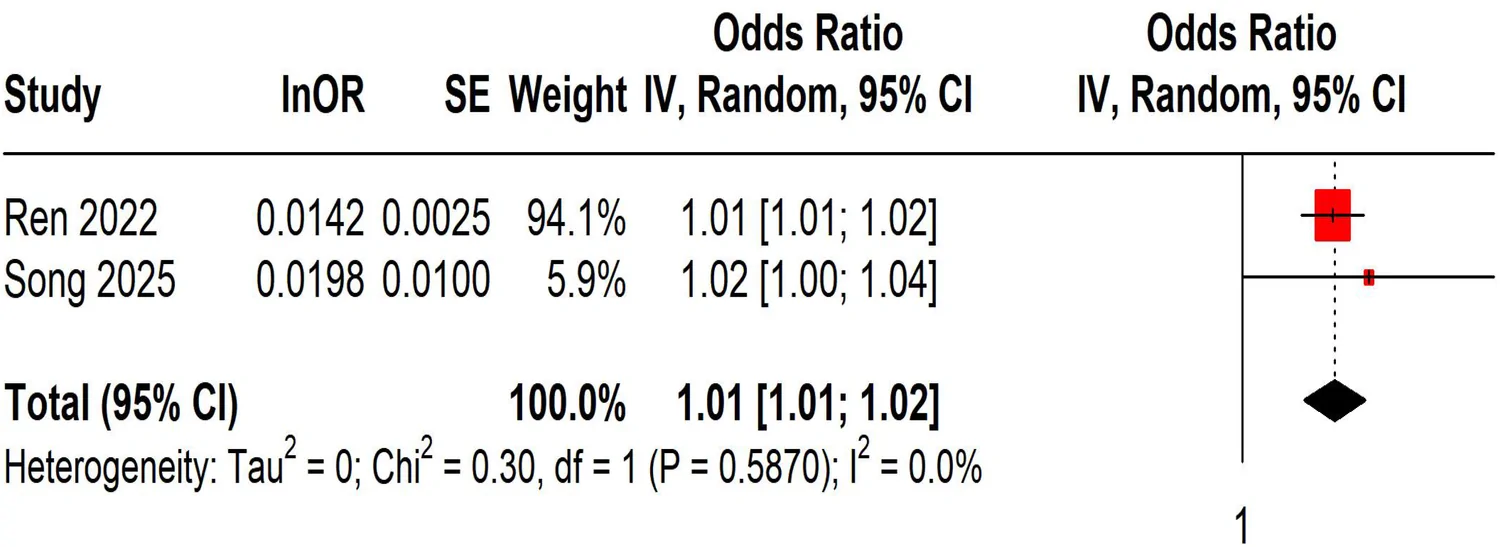
Primary continuous-NLR OR meta-analysis (Ren 2022 + song 2025, k = 2; wang 2024 was EXCLUDED because of internally inconsistent OR/*β* values, and zhao 2020 was EXCLUDED because it does not report a directly extractable continuous per-unit OR).

**Figure 4:**
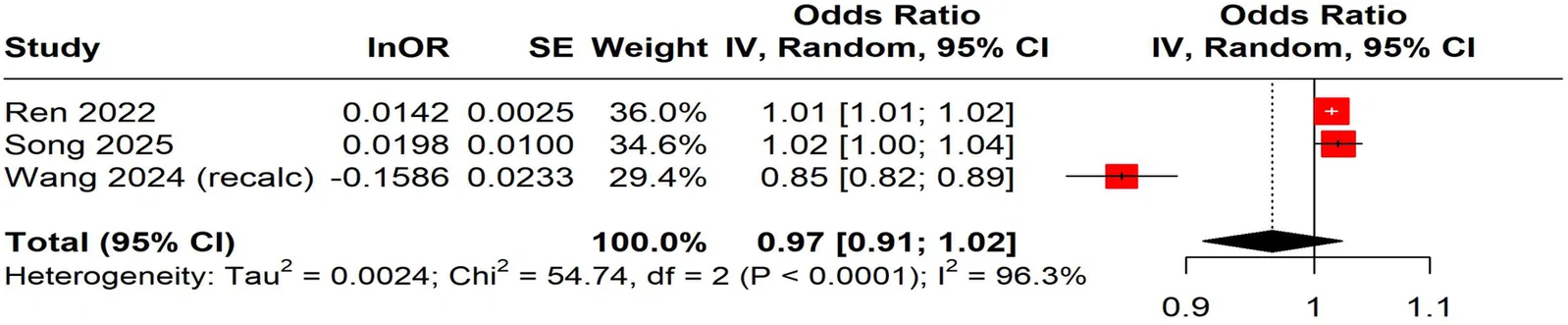
Exploratory sensitivity analysis — continuous NLR OR with wang 2024-recalc (Ren 2022 + song 2025 + wang 2024-recalc, k = 3).

### 3.4 PCT and mortality or adverse prognosis

Two studies reported OR for PCT in relation to mortality or short-term adverse prognosis, but per §2.5 mortality and composite adverse-prognosis endpoints are not pooled. Wang 2024 (mortality endpoint, OR = 1.957, 95% CI 1.243–3.081) and Li Liang 2024 (composite short-term adverse-prognosis endpoint, OR = 2.827, 95% CI 1.694–4.718) are therefore reported narratively; Figure 5 shows endpoint-specific estimates without a pooled diamond.

**Figure 5:**
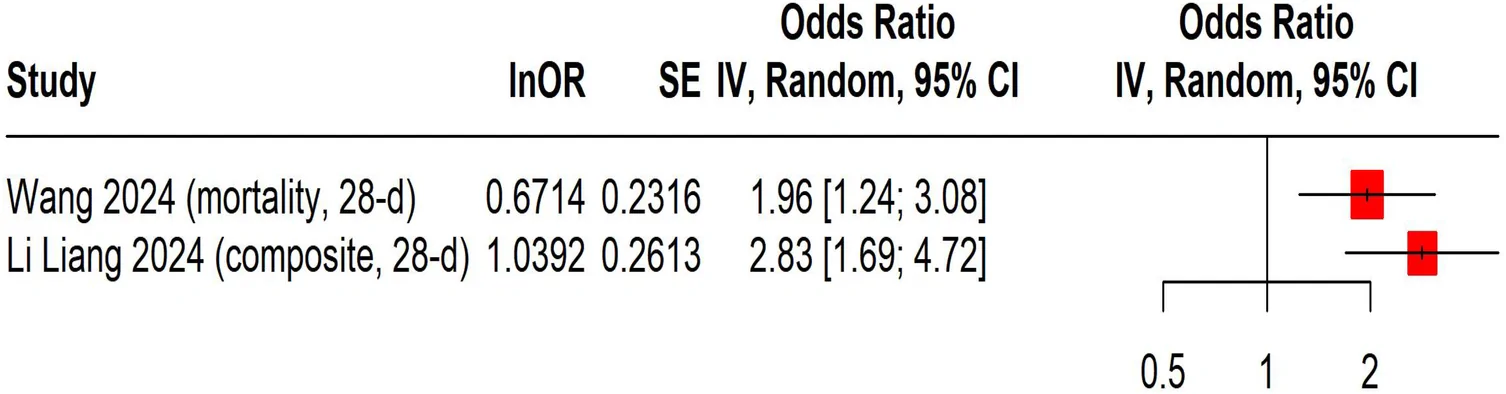
PCT forest plot—categorical PCT (high vs. low) odds ratios from the two contributing studies. Endpoint-specific estimates only: Wang 2024 (mortality endpoint, OR = 1.957, 95% CI 1.243−3.081) and Li Liang 2024 (composite adverse-prognosis endpoint, OR = 2.827, 95% CI 1.694−4.718). Per §2.5, mortality and composite adverse-prognosis endpoints are not pooled; no pooled diamond is shown.

### 3.5 SII and mortality

SII showed a relatively consistent association with mortality (see Figure 6 for forest plot).

**Figure 6:**
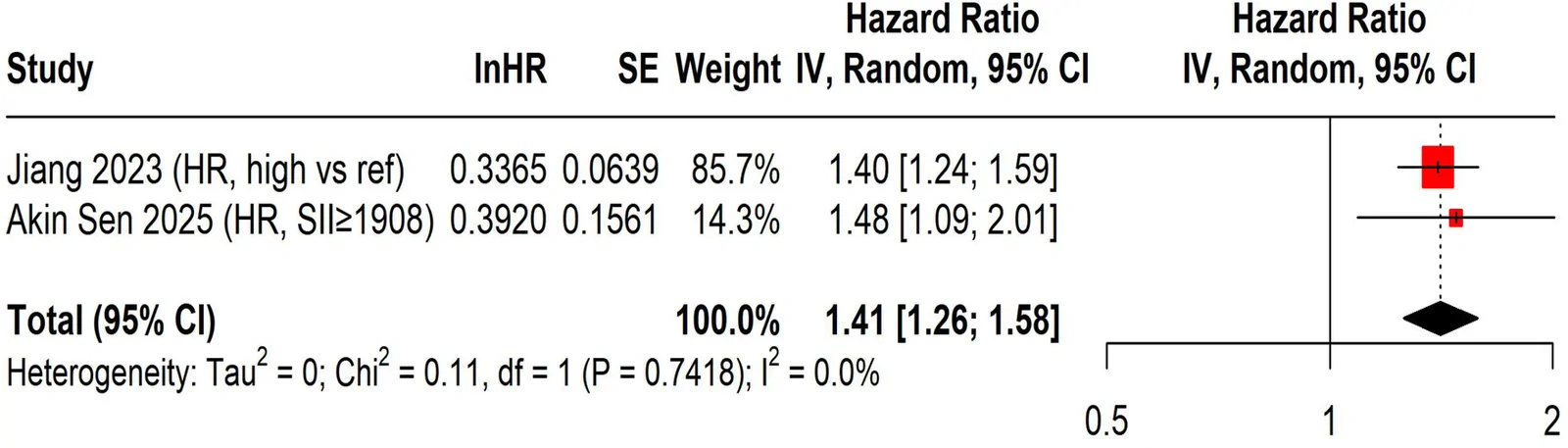
SII and mortality Figure 6. SII and mortality — forest plot of adjusted hazard ratios for high vs. low SII (Jiang 2023 and Akin Sen 2025 pooled, k = 2; DerSimonian–Laird random-effects model).

### 3.6 PLR and mortality

Categorical PLR thresholds were reported by only two studies (see Figure 7 for forest plot). The corrected quantitative synthesis indicates that PCT and SII have the consistent prognostic signals. NLR has possible prognostic value but is unstable across studies. PLR currently lacks robust pooled evidence. The most important methodological finding is that effect estimates should not be pooled indiscriminately across OR, HR, continuous effects, categorical effects, and different mortality outcomes. The apparent prognostic value of a biomarker can change materially depending on how the biomarker is modeled and which effect measure is reported.

**Figure 7:**
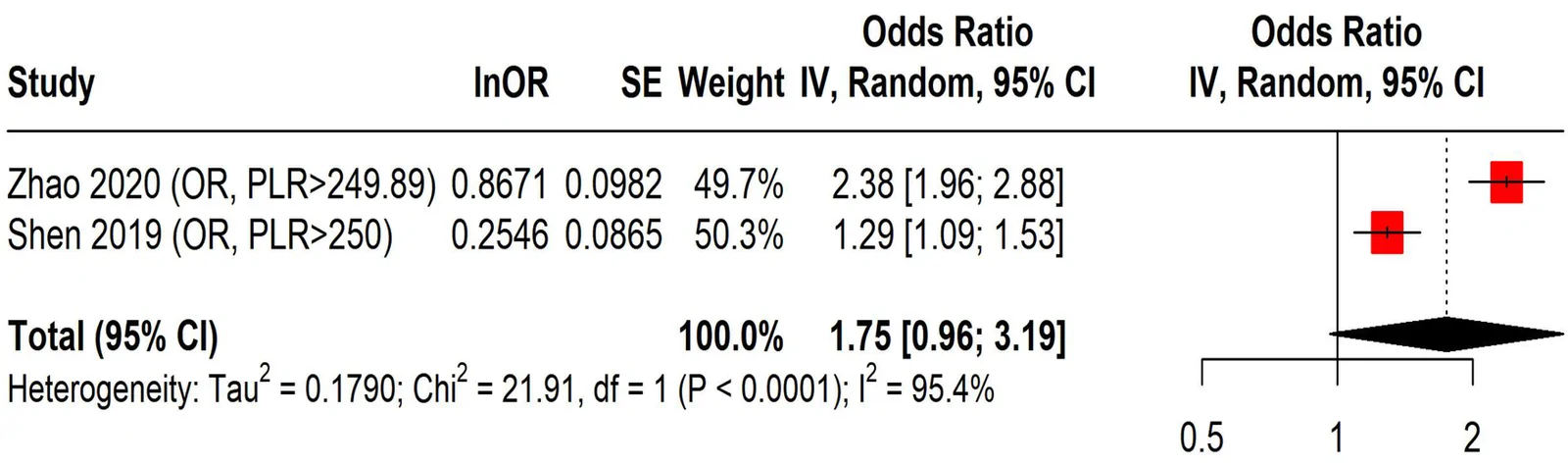
PLR and mortality in sepsis/septic shock — forest plot of categorical PLR odds ratios (zhao 2020 and shen 2019 pooled, k = 2; derSimonian–laird random-effects model).

## 4 Discussion

### 4.1 Principal findings

This systematic review and meta-analysis provides a more cautious and statistically coherent interpretation of inflammatory biomarkers in sepsis prognosis. Regarding NLR specifically, the interpretation depends on the analytic framework: (i) the revised primary continuous-NLR OR analysis (k = 2, Ren 2022 + Song 2025) yields OR = 1.0146 (95% CI 1.0098–1.0195; *P* < 0.001) with I^2^ = 0.0%, indicating minimal heterogeneity and a small but statistically robust positive association; (ii) the exploratory k = 3 sensitivity analysis including the unverified Wang 2024-recalc value yields OR = 0.9658 (95% CI 0.9118–1.0230; *P* = 0.236) with I^2^ = 96.4%, reflecting the influence of the Wang 2024 value on heterogeneity; (iii) the HR-based analysis of Li X 2024 and Song 2025 yields HR = 1.02 (95% CI 1.01–1.03) with I^2^ = 34.9%. Earlier reports of high heterogeneity (I^2^ = 97.0%) reflected the 4-study pooled analysis that is no longer the primary analytic framework. PLR currently lacks robust pooled evidence.

### 4.2 Comparison with previous literature

Previous studies have linked NLR, PLR, SII, and PCT with outcomes in infection, cardiovascular disease, malignancy, and critical illness (6–17, 31–40). In sepsis, however, the immune response is dynamic. Early hyperinflammation may coexist with or progress into immunosuppression. Biomarkers measured at a single time point may therefore reflect only one phase of a changing process. Several recent studies have emphasized the value of combined models rather than single biomarkers (18, 20, 21, 23, 24). This direction is clinically reasonable. A single biomarker is unlikely to capture the full complexity of sepsis. Combining biomarkers with severity scores, lactate, organ function markers, and comorbidities may improve prognostic accuracy.

### 4.3 Implications for research

Given the high heterogeneity, small number of studies, and PROSPERO protocol deviations documented above, the present meta-analysis does not support specific clinical recommendations for the use of NLR, PLR, PCT, or SII in routine sepsis prognostication. The available evidence is insufficient to justify changes to existing clinical practice, and biomarker-based risk stratification should remain an adjunct to (rather than a replacement for) established clinical judgment, severity scoring (e.g., SOFA, APACHE II), and standard-of-care monitoring. Future research should: (1) standardize biomarker assay platforms and reporting (including assay manufacturer, cut-off selection method, and timing of measurement); (2) pre-register analyses with explicit outcome definitions; (3) report effect estimates by biomarker modeling form (continuous vs. categorical) and effect measure (OR vs. HR) separately; (4) include multi-center, multi-ethnic cohorts; and (5) follow the PROSPERO protocol without *post-hoc* deviations. Until such evidence accumulates, the present findings should be treated as hypothesis-generating only.

### 4.4 Limitations

This study has several limitations that should be acknowledged. FIRST AND FOREMOST, this meta-analysis deviates substantially from its pre-registered PROSPERO protocol (CRD420251035190, Version 1.1, 7 September 2026) in three core dimensions: the research question was changed from sepsis-onset prediction to mortality prognostication in established sepsis; the registered biomarkers (NLR, PLR, dNLR, LMR, SII) were modified to NLR, PLR, PCT, SII; and the registered HR-only effect measure was expanded to include OR. Because these deviations change the core research question, the biomarker set, and the analytic approach, the present synthesis should be interpreted with caution: the pooled estimates are not directly comparable to any pre-specified effect, and the strength of evidence is inherently weaker than that of a protocol-conforming meta-analysis. These deviations are documented in PROSPERO Version 1.1 and in Supplementary Table S2. Third, the modest number of included studies (*n* = 10) and relatively small total sample size (*N* = 2,412) limit the statistical power of some analyses, particularly for PLR and SII where only 2 studies were available.

Fourth, during eligibility assessment, two pairs of included studies were examined for potential cohort overlap. Pair A (Xicong Li 2024 and Yinlong Ren 2022) and Pair B (Daishan Jiang 2023 and Tong Wang 2024) were flagged because the original reports did not provide sufficient detail (e.g., database version, recruitment period, and institution) to definitively exclude shared patients. For each pair, the study with the larger sample size and more complete outcome reporting was retained for the corresponding quantitative synthesis; sensitivity analyses excluding the smaller study in each pair produced pooled estimates that were not materially different in direction or magnitude (Supplementary Table S5).

Fifth, outcomes were heterogeneous and included ICU mortality, in-hospital mortality, 28-day mortality, and adverse prognosis. These are clinically different endpoints, and per §2.5 the primary synthesis did not pool mortality with the composite short-term adverse-prognosis outcome. One residual cross-endpoint display remains in §3.4 Figure 5, which is explicitly labelled as not a mortality-specific effect because one of the two contributing studies is a composite-endpoint study; future updates should stratify by endpoint (all-cause mortality vs. composite short-term adverse prognosis) when additional studies become available. The present synthesis also did not address sepsis-associated acute respiratory distress syndrome (ARDS) as a distinct subgroup, although NLR and SII have been evaluated for prognostic value in this population (41). Sixth, biomarker thresholds were often data-derived within cohorts, limiting cross-study comparability. Seventh, adjustment models varied substantially across studies. Eighth, formal publication bias testing was not appropriate because each subgroup included fewer than 10 studies.

### 4.5 Methodological implications

This review highlights several methodological issues relevant to future biomarker research. First, continuous and categorical effects should be reported clearly and separately. Second, authors should report whether OR, HR,*β* coefficients, or standard errors are being presented. The Wang 2024 table illustrates how mislabeling can affect meta-analysis and interpretation. Third, biomarker thresholds should not be derived and validated in the same cohort without external validation. Fourth, adjustment models should include key confounders such as age, SOFA or APACHE II score, infection source, renal function, vasopressor use, and mechanical ventilation. Fifth, studies should report model performance and incremental value over established severity scores.

### 4.6 Conclusions

This systematic review and meta-analysis suggests that PCT and SII have the consistent prognostic signals for mortality or adverse short-term prognosis in patients with sepsis and septic shock. NLR may have prognostic value, but the evidence is unstable and highly heterogeneous. PLR does not currently show a robust pooled association with mortality. These biomarkers should be interpreted as exploratory components of multidimensional risk assessment rather than independent decision-making tools. Future prospective multicenter studies should standardize measurement timing, biomarker thresholds, outcome definitions, and adjustment strategies to clarify their role in clinical prediction models.

## Data Availability

The datasets presented in this study can be found in online repositories. The names of the repository/repositories and accession number(s) can be found in the article/Supplementary Material.
