# Supplementary material for: Prognostic value of NLR, SII, PCT, and PLR in sepsis and septic shock: a systematic review and meta-analysis
Source: Front Med (Lausanne). 2026 Sep 25;13:1893289. doi: 10.3389/fmed.2026.1893289 (PMC13649752; doi:10.3389/fmed.2026.1893289)

Study	lnOR	SE	Weight	Odds Ratio IV, Random, 95% CI	Odds Ratio IV, Random, 95% CI
Ren 2022	0.0142	0.0025	94.1%	1.01 [1.01; 1.02]	
Song 2025	0.0198	0.0100	5.9%	1.02 [1.00; 1.04]	
**Total (95% CI)**			**100.0%**	**1.01 [1.01; 1.02]**	

Heterogeneity: $Tau^2 = 0$; $Chi^2 = 0.30$, df = 1 (P = 0.5870); $I^2 = 0.0\%$

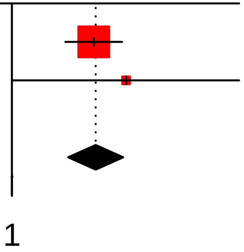

Supplement: Supplementary file 1 [file Datasheet1.zip › Figures/Fig3_NLR_primary_r5.pdf]
