# Supplementary material for: Prognostic value of NLR, SII, PCT, and PLR in sepsis and septic shock: a systematic review and meta-analysis
Source: Front Med (Lausanne). 2026 Sep 25;13:1893289. doi: 10.3389/fmed.2026.1893289 (PMC13649752; doi:10.3389/fmed.2026.1893289)

Study	lnOR	SE	Weight	Odds Ratio IV, Random, 95% CI
Ren 2022	0.0142	0.0025	36.0%	1.01 [1.01; 1.02]
Song 2025	0.0198	0.0100	34.6%	1.02 [1.00; 1.04]
Wang 2024 (recalc)	−0.1586	0.0233	29.4%	0.85 [0.82; 0.89]
**Total (95% CI)**			**100.0%**	**0.97 [0.91; 1.02]**

Heterogeneity: $Tau^2 = 0.0024$; $Chi^2 = 54.74$, df = 2 (P < 0.0001); $I^2 = 96.3\%$

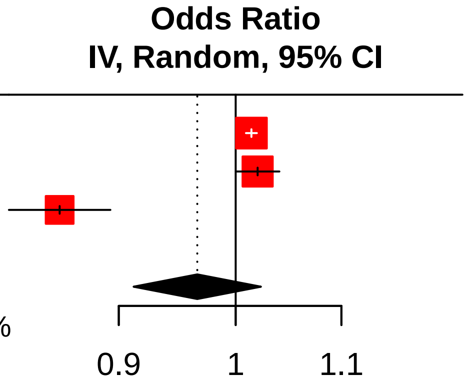

Supplement: Supplementary file 1 [file Datasheet1.zip › Figures/Fig4_NLR_exploratory_r5.pdf]
