# Supplementary material for: Prognostic value of NLR, SII, PCT, and PLR in sepsis and septic shock: a systematic review and meta-analysis
Source: Front Med (Lausanne). 2026 Sep 25;13:1893289. doi: 10.3389/fmed.2026.1893289 (PMC13649752; doi:10.3389/fmed.2026.1893289)

Study	lnOR	SE	Odds Ratio IV, Random, 95% CI
Wang 2024 (mortality, 28−d)	0.6714	0.2316	1.96 [1.24; 3.08]
Li Liang 2024 (composite, 28−d)	1.0392	0.2613	2.83 [1.69; 4.72]

Odds Ratio
IV, Random, 95% CI

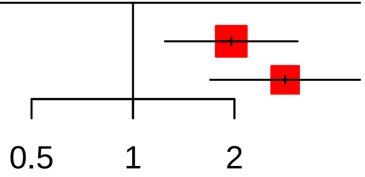

Supplement: Supplementary file 1 [file Datasheet1.zip › Figures/Fig5_PCT_endpoints_r5.pdf]
