# Supplementary material for: Prognostic value of NLR, SII, PCT, and PLR in sepsis and septic shock: a systematic review and meta-analysis
Source: Front Med (Lausanne). 2026 Sep 25;13:1893289. doi: 10.3389/fmed.2026.1893289 (PMC13649752; doi:10.3389/fmed.2026.1893289)

Study	lnHR	SE	Weight	Hazard Ratio IV, Random, 95% CI
Jiang 2023 (HR, high vs ref)	0.3365	0.0639	85.7%	1.40 [1.24; 1.59]
Akin Sen 2025 (HR, SII=1908)	0.3920	0.1561	14.3%	1.48 [1.09; 2.01]
**Total (95% CI)**			**100.0%**	**1.41 [1.26; 1.58]**

Heterogeneity: $Tau^2 = 0$; $Chi^2 = 0.11$, df = 1 (P = 0.7418); $I^2 = 0.0\%$

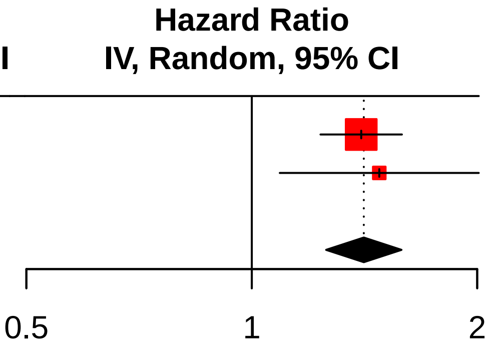

Supplement: Supplementary file 1 [file Datasheet1.zip › Figures/Fig6_SII_HR_r5.pdf]
