# Supplementary material for: Prognostic value of NLR, SII, PCT, and PLR in sepsis and septic shock: a systematic review and meta-analysis
Source: Front Med (Lausanne). 2026 Sep 25;13:1893289. doi: 10.3389/fmed.2026.1893289 (PMC13649752; doi:10.3389/fmed.2026.1893289)

Study	lnOR	SE	Weight	Odds Ratio IV, Random, 95% CI
Zhao 2020 (OR, PLR>249.89)	0.8671	0.0982	49.7%	2.38 [1.96; 2.88]
Shen 2019 (OR, PLR>250)	0.2546	0.0865	50.3%	1.29 [1.09; 1.53]
**Total (95% CI)**			**100.0%**	**1.75 [0.96; 3.19]**

Heterogeneity: $Tau^2 = 0.1790$; $Chi^2 = 21.91$, df = 1 ($P < 0.0001$); $I^2 = 95.4\%$

Odds Ratio
IV, Random, 95% CI

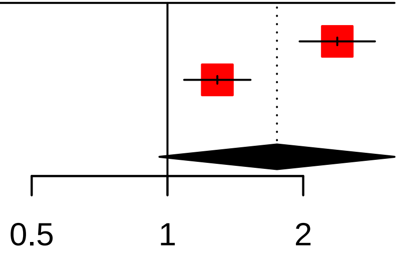

Supplement: Supplementary file 1 [file Datasheet1.zip › Figures/Fig7_PLR_OR_r5.pdf]
