# Supplementary material for: Prognostic value of NLR, SII, PCT, and PLR in sepsis and septic shock: a systematic review and meta-analysis
Source: Front Med (Lausanne). 2026 Sep 25;13:1893289. doi: 10.3389/fmed.2026.1893289 (PMC13649752; doi:10.3389/fmed.2026.1893289)
Supplement: Supplementary file 1 [file Datasheet1.zip › Supplementary_Tables/Supplementary_Table_2_Risk_of_Bias.docx]

**Supplementary Table 2. Risk-of-Bias Assessment**

Risk of bias was summarized using PROBAST-informed domains and full-text information from the included studies. Domains included participant selection, predictor measurement, outcome definition, and statistical analysis.

| **Study** | **Participants** | **Predictors** | **Outcomes** | **Analysis** | **Overall concern** | **Main reason** |
| --- | --- | --- | --- | --- | --- | --- |
| Li 2024 J Emerg Med | Moderate | Low | Low | Moderate | Moderate | Retrospective septic shock cohort; SII and PCT were objective laboratory markers, but model development, threshold handling, and external validation were limited. |
| Ren 2022 BMC Pulm Med | Moderate | Low | Low | Moderate | Moderate | Retrospective MIMIC-III database study; NLR was routinely measured, but database coding, missing data, and residual confounding may affect estimates. |
| Wang 2024 Zhonghua | Moderate | Low | Low | Moderate | Moderate | Retrospective multicenter emergency septic shock study; PCT and NLR were available, but one NLR regression row required recalculation from beta and confidence interval. |
| Zhao 2020 Int Immunopharmacol | Moderate | Low | Low | Moderate | Moderate | Large MIMIC-III cohort; NLR and PLR were objectively calculated, but biomarker thresholds were data-derived and require external validation. |
| Shen 2019 BMJ Open | Moderate | Low | Low | Moderate | Moderate | Retrospective database study; PLR was objective, but disease-severity interaction, threshold selection, and residual confounding limit certainty. |
| Jiang 2023 Clin Exp Med | Moderate | Low | Low | Moderate | Moderate | MIMIC-IV database study; SII was objectively calculated, but external validation was limited and residual confounding remains possible. |
| Zhou 2024 Crit Rev Immunol | Moderate | Low | Low | High | High | No adjusted mortality OR or HR was available for primary pooling; only a crude SII effect could be calculated from reported counts. |
| Li 2024 BMC Infect Dis | Moderate | Low | Low | Moderate | Moderate | Retrospective MIMIC-IV study in sepsis with coronary artery disease; the specific comorbidity profile limits applicability to general sepsis populations. |
| Song 2025 BMC Gastroenterol | Moderate | Low | Low | Moderate | Moderate | Retrospective MIMIC-IV study in sepsis with liver cirrhosis; liver disease may affect blood-cell indices and limits generalizability. |
| Akin Sen 2025 Medicine | Moderate | Low | Low | Moderate | Moderate | Retrospective ICU study in patients aged 85 years or older; very elderly population introduces indirectness for general adult sepsis. |

Domain definitions:

- Participants: selection of study population and representativeness.
- Predictors: definition, timing, and measurement of NLR, PLR, SII, and PCT.
- Outcomes: mortality or short-term adverse prognosis definition and ascertainment.
- Analysis: adjustment, missing data, model specification, threshold handling, and validation.

Predictor measurement was generally judged as low concern because NLR, PLR, SII, and PCT are laboratory-based markers. The analysis domain was the main source of concern because many studies were retrospective, used data-derived thresholds, lacked external validation, or did not consistently adjust for severity scores such as SOFA or APACHE II.
