# Supplementary material for: Prognostic value of NLR, SII, PCT, and PLR in sepsis and septic shock: a systematic review and meta-analysis
Source: Front Med (Lausanne). 2026 Sep 25;13:1893289. doi: 10.3389/fmed.2026.1893289 (PMC13649752; doi:10.3389/fmed.2026.1893289)
Supplement: Supplementary file 1 [file Datasheet1.zip › Supplementary_Tables/Supplementary_Table_3_Study_Characteristics.docx]

**Supplementary Table 3. Characteristics of Included Studies**

This table was reconstructed from the extracted dataset and manuscript text. It should be checked against the original full texts before submission.

| **Study** | **PMID** | **PMCID** | **DOI** | **Country** | **Design/data source** | **Population** | **Sample size** | **Biomarker(s) used in synthesis** | **Outcome(s)** | **Effect measure** |
| --- | --- | --- | --- | --- | --- | --- | --- | --- | --- | --- |
| Li 2024 J Emerg Med | 39183119 |  | 10.1016/j.jemermed.2024.05.005 | China | Retrospective | Septic shock patients | 200 | SII, PCT | 28-day adverse prognosis | OR |
| Ren 2022 BMC Pulm Med | 34991569 | PMC8739695 | 10.1186/s12890-021-01809-8 | USA | Retrospective MIMIC-III | ICU patients with sepsis and lung infection | 1,676 | NLR | In-hospital mortality | OR |
| Wang 2024 Zhonghua | 38991955 |  | 10.3760/cma.j.cn121430-20230703-00486 | China | Retrospective multicenter | Emergency department septic shock patients | 860 | NLR, PCT | 28-day mortality | OR |
| Zhao 2020 Int Immunopharmacol | 31955067 |  | 10.1016/j.intimp.2019.106145 | China | MIMIC-III database analysis | Septic patients admitted to ICU from the emergency department | 5,663 | NLR, PLR | 28-day mortality | OR |
| Shen 2019 BMJ Open | 30782690 | PMC6352809 | 10.1136/bmjopen-2018-022896 | China | MIMIC-III database analysis | Sepsis patients | 5,537 | PLR | ICU mortality | OR |
| Jiang 2023 Clin Exp Med | 36930382 |  | 10.1007/s10238-023-01029-w | USA | Retrospective MIMIC-IV | Critically ill patients with sepsis | 16,007 | SII | 28-day mortality | HR |
| Zhou 2024 Crit Rev Immunol | 38848289 |  | 10.1615/CritRevImmunol.2024051413 | China | Observational | Sepsis patients grouped by severity | 183 | SII | 28-day mortality | Crude OR calculated |
| Li 2024 BMC Infect Dis | 39075364 | PMC11288105 | 10.1186/s12879-024-09516-5 | China | Retrospective MIMIC-IV | Sepsis patients with coronary artery disease | 1,175 | NLR, PLR | 28-day mortality | HR |
| Song 2025 BMC Gastroenterol | 40751177 | PMC12315451 | 10.1186/s12876-025-04157-7 | China | Retrospective MIMIC-IV | Sepsis patients with liver cirrhosis | 1,372 | NLR | In-hospital and 28-day mortality | OR, HR |
| Akin Sen 2025 Medicine | 41189164 | PMC12537170 | 10.1097/MD.0000000000044979 | Turkey | Retrospective | ICU sepsis patients aged 85 years or older | 381 | NLR, SII, PLR | ICU/28-day mortality | HR |

Note: PubMed identifiers were verified through NCBI E-utilities. Sample sizes were taken from the final extraction dataset.
