# Supplementary material for: Prognostic value of NLR, SII, PCT, and PLR in sepsis and septic shock: a systematic review and meta-analysis
Source: Front Med (Lausanne). 2026 Sep 25;13:1893289. doi: 10.3389/fmed.2026.1893289 (PMC13649752; doi:10.3389/fmed.2026.1893289)
Supplement: Supplementary file 1 [file Datasheet1.zip › Supplementary_Tables/Supplementary_Table_4_Certainty_of_Evidence.docx]

**Supplementary Table 4. Certainty of Evidence**

Certainty was summarized narratively using GRADE-consistent domains because the included evidence came from observational prognostic studies and most quantitative subgroups included very few studies.

| **Biomarker synthesis** | **Studies** | **Main result** | **Risk of bias** | **Inconsistency** | **Indirectness** | **Imprecision** | **Publication bias** | **Overall certainty** |
| --- | --- | --- | --- | --- | --- | --- | --- | --- |
| PCT, OR scale | 2 | Pooled OR 2.3057, 95% CI 1.6113-3.2993 | Serious | Not serious | Serious | Serious | Undetected; k < 10 | Low |
| SII, HR scale | 2 | Pooled HR 1.4112, 95% CI 1.2568-1.5846 | Serious | Not serious | Serious | Serious | Undetected; k < 10 | Low |
| NLR, OR scale | 4 | Pooled OR 0.9925, 95% CI 0.9586-1.0277 | Serious | Very serious | Serious | Serious | Undetected; k < 10 | Very low |
| NLR, HR scale | 2 | Pooled HR 1.0176, 95% CI 1.0092-1.0260 | Serious | Serious | Serious | Serious | Undetected; k < 10 | Low to very low |
| PLR, categorical OR scale | 2 | Pooled OR 1.7491, 95% CI 0.9597-3.1877 | Serious | Very serious | Serious | Serious | Undetected; k < 10 | Very low |

The certainty labels are conservative because the included evidence came from observational prognostic studies and most quantitative subgroups included very few studies.
