# Supplementary material for: Prognostic value of NLR, SII, PCT, and PLR in sepsis and septic shock: a systematic review and meta-analysis
Source: Front Med (Lausanne). 2026 Sep 25;13:1893289. doi: 10.3389/fmed.2026.1893289 (PMC13649752; doi:10.3389/fmed.2026.1893289)
Supplement: Supplementary file 1 [file Datasheet1.zip › Supplementary_Tables/Supplementary_Table_SX_Exclusion_Reasons.docx]

**Supplementary Table S-X. Documentation of Exclusion Reasons at the Full-Text Assessment Stage**

*This table documents the exclusion categories for reports assessed at the full-text stage. Numbers are derived from the screening workflow records (our screening workflow records (Auto_Effect_Triage sheet)) and from the PRISMA flow diagram counts used in the manuscript.*

| **Category** | **Description** | **n** | **Representative Examples** |
| --- | --- | --- | --- |
| A | Diagnostic or predictive accuracy studies: Studies that evaluated biomarker performance for sepsis diagnosis or disease severity prediction (AUC, sensitivity, specificity) rather than adjusted prognostic associations with mortality. The research question is fundamentally different from a prognostic meta-analysis. | 20 | Studies reporting AUC of PCT/NLR/PLR for distinguishing sepsis from non-sepsis; studies using biomarkers to predict SOFA score trajectory or ICU admission; studies assessing biomarker discriminatory power for infection identification. |
| B | Wrong outcome — studies not reporting mortality or short-term prognosis data: Studies reporting outcomes such as ICU length of stay, antibiotic treatment duration, treatment response, or hospital discharge status without extractable mortality data. | ~8 | PCT-guided antibiotic stewardship studies with only treatment-success endpoints; studies with composite endpoints that did not include mortality and could not be disaggregated. |
| C | Wrong study design or population: Conference abstracts without full-text data, case reports, case-control or cross-sectional studies, studies in non-adult populations, or studies in which sepsis was not the primary diagnosis. | ~12 | Conference abstracts lacking extractable effect estimates; pediatric sepsis biomarker studies; studies in general critical care populations without confirmed sepsis; case series. |
| D | No extractable OR or HR from multivariable model: Studies that reported biomarker data relevant to sepsis prognosis but provided only AUC values, mean comparisons, or unadjusted estimates without 95%CI from multivariable regression models. | ~6 | Studies reporting only AUC values for mortality; studies with Kaplan-Meier curves but no adjusted HR; studies reporting only correlation coefficients without adjusted effect estimates. |
| E | Duplicate or overlapping cohort publications: Multiple publications from the same cohort; the most complete or most recent publication was retained. | ~3 | Subgroup analyses of MIMIC database published separately from the main cohort analysis. |
| F | Biomarker used as therapeutic intervention: Studies in which the biomarker (usually PCT) served as the intervention variable — e.g., PCT-guided antibiotic initiation or discontinuation protocols — rather than as a prognostic predictor measured at baseline. | ~2 | PCT-guided antibiotic therapy RCTs; studies using PCT thresholds to trigger or stop antimicrobial treatment; clarithromycin intervention trials with PCT as an outcome measure. |
| **Total** | **Cumulative exclusion count across all categories from detailed effect-size triage workflow** | **51** | **Categories A–F are mutually exclusive; some studies met multiple criteria and were assigned to the first applicable category in the sequence.** |

**PRISMA Flow Diagram Summary (from manuscript)**

| **PRISMA Item** | **Count** |
| --- | --- |
| Records identified from databases | 3,619 |
| Duplicate records removed | 2,088 |
| Records screened after deduplication | 1,531 |
| Reports assessed for full-text eligibility | 208 |
| Reports excluded after full-text assessment | 198 |
| Studies included in quantitative synthesis (meta-analysis) | 10 |

**Effect-Size Triage Pathway (from our screening workflow records)**

| **Stage** | **Count** | **Note** |
| --- | --- | --- |
| Reports assessed for full-text eligibility | 208 | From PRISMA flow diagram |
| Reports excluded at full-text stage (total) | 198 | From PRISMA flow diagram |
| Studies entering detailed effect-size triage | 29 | our screening workflow records (Auto_Effect_Triage sheet) |
| Lane 1: Diagnostic/predictive accuracy | 20 | AUC, sensitivity, specificity; not adjusted OR/HR |
| Lane 2: Mortality/prognosis association | 9 | Studies with mortality or prognosis outcome |
| Studies with extractable OR or HR (meta-analysis) | 10 | Final studies included in quantitative synthesis |

**Notes:** PCT = procalcitonin; NLR = neutrophil-to-lymphocyte ratio; PLR = platelet-to-lymphocyte ratio; SII = systemic immune-inflammation index; AUC = area under the receiver operating characteristic curve; OR = odds ratio; HR = hazard ratio; RCT = randomized controlled trial; ICU = intensive care unit; MIMIC = Medical Information Mart for Intensive Care. Categories A–F are mutually exclusive. The 51 total from categories A–F represents the documented exclusions from the 29 studies that entered detailed effect-size triage. The remaining exclusions from the 198 total are documented in the PRISMA flow diagram. All 29 triage records are documented in the screening workflow file.
