# Supplementary material for: Prognostic value of NLR, SII, PCT, and PLR in sepsis and septic shock: a systematic review and meta-analysis
Source: Front Med (Lausanne). 2026 Sep 25;13:1893289. doi: 10.3389/fmed.2026.1893289 (PMC13649752; doi:10.3389/fmed.2026.1893289)
Supplement: Supplementary file 1 [file Datasheet1.zip › Supplementary_Tables/Supplementary_File_1_Search_Strategy.docx]

**Supplementary File 1. Search Strategy**

This file reports the search strategy used for the systematic review. Database-specific syntax was adapted as appropriate for each source.

# Databases

PubMed, MEDLINE, EMBASE, Web of Science, Cochrane Library, CNKI, Wanfang, VIP, and Chinese Biomedical Literature Database were searched from inception to December 22, 2025.

# Core English Search Terms

The search combined sepsis terms, prognosis terms, and inflammatory biomarker terms.

(sepsis OR septic shock)
AND
(mortality OR death OR prognosis OR prognostic OR prediction OR "prediction model" OR outcome)
AND
("neutrophil-to-lymphocyte ratio" OR NLR OR "platelet-to-lymphocyte ratio" OR PLR OR "systemic immune-inflammation index" OR SII OR procalcitonin OR PCT)

# PubMed Strategy

("Sepsis"[Mesh] OR sepsis[Title/Abstract] OR "septic shock"[Title/Abstract])
AND
(mortality[Title/Abstract] OR death[Title/Abstract] OR prognosis[Title/Abstract] OR prognostic[Title/Abstract] OR prediction[Title/Abstract] OR "prediction model"[Title/Abstract] OR outcome[Title/Abstract])
AND
("neutrophil-to-lymphocyte ratio"[Title/Abstract] OR NLR[Title/Abstract] OR "platelet-to-lymphocyte ratio"[Title/Abstract] OR PLR[Title/Abstract] OR "systemic immune-inflammation index"[Title/Abstract] OR SII[Title/Abstract] OR procalcitonin[Title/Abstract] OR PCT[Title/Abstract])

# Embase / MEDLINE Strategy

('sepsis'/exp OR sepsis:ti,ab OR 'septic shock':ti,ab)
AND
(mortality:ti,ab OR death:ti,ab OR prognosis:ti,ab OR prognostic:ti,ab OR prediction:ti,ab OR 'prediction model':ti,ab OR outcome:ti,ab)
AND
('neutrophil to lymphocyte ratio':ti,ab OR nlr:ti,ab OR 'platelet to lymphocyte ratio':ti,ab OR plr:ti,ab OR 'systemic immune inflammation index':ti,ab OR sii:ti,ab OR procalcitonin:ti,ab OR pct:ti,ab)

# Web of Science Strategy

TS=(sepsis OR "septic shock")
AND TS=(mortality OR death OR prognosis OR prognostic OR prediction OR "prediction model" OR outcome)
AND TS=("neutrophil-to-lymphocyte ratio" OR NLR OR "platelet-to-lymphocyte ratio" OR PLR OR "systemic immune-inflammation index" OR SII OR procalcitonin OR PCT)

# Cochrane Library Strategy

(sepsis OR "septic shock")
AND
(mortality OR death OR prognosis OR prognostic OR prediction OR "prediction model" OR outcome)
AND
("neutrophil-to-lymphocyte ratio" OR NLR OR "platelet-to-lymphocyte ratio" OR PLR OR "systemic immune-inflammation index" OR SII OR procalcitonin OR PCT)

# Chinese Database Strategy

Chinese databases included CNKI, Wanfang, VIP, and Chinese Biomedical Literature Database.

(脓毒症 OR 感染性休克 OR 脓毒性休克)
AND
(死亡 OR 预后 OR 预测 OR 预测模型 OR 不良预后)
AND
(中性粒细胞淋巴细胞比值 OR 中性粒细胞/淋巴细胞比值 OR NLR OR 血小板淋巴细胞比值 OR 血小板/淋巴细胞比值 OR PLR OR 系统免疫炎症指数 OR SII OR 降钙素原 OR PCT)

# Manual Searching

Reference lists of relevant articles and included studies were manually screened to identify additional eligible records.
