# Supplementary material for: Prognostic value of NLR, SII, PCT, and PLR in sepsis and septic shock: a systematic review and meta-analysis
Source: Front Med (Lausanne). 2026 Sep 25;13:1893289. doi: 10.3389/fmed.2026.1893289 (PMC13649752; doi:10.3389/fmed.2026.1893289)
Supplement: Supplementary file 1 [file Datasheet1.zip › Supplementary_Tables/Supplementary_File_2_PubMed_Search_and_Verification.docx]

**Supplementary File 2. PubMed Search and Included-Study Verification**

This file documents the PubMed-only verification step. Other databases were not re-audited in this step.

# PubMed Search Count Checks

The following PubMed searches were queried through NCBI E-utilities.

| **Strategy** | **Search date** | **Date limit** | **Count** |
| --- | --- | --- | --- |
| Broad PubMed strategy combining sepsis, prognosis, and NLR/PLR/SII/PCT terms | 2026-05-28 | Inception to 2025-12-22 | 3,089 |
| Exact-phrase biomarker strategy combining sepsis, prognosis, and NLR/PLR/SII/PCT exact phrases | 2026-05-28 | Inception to 2025-12-22 | 2,254 |

These counts should not be used as the total PRISMA count because the manuscript also searched non-PubMed databases.

# Verified PubMed Records for Included Studies

| **Study** | **PMID** | **PMCID** | **DOI** | **Journal** |
| --- | --- | --- | --- | --- |
| Li 2024 J Emerg Med | 39183119 |  | 10.1016/j.jemermed.2024.05.005 | The Journal of Emergency Medicine |
| Ren 2022 BMC Pulm Med | 34991569 | PMC8739695 | 10.1186/s12890-021-01809-8 | BMC Pulmonary Medicine |
| Wang 2024 Zhonghua | 38991955 |  | 10.3760/cma.j.cn121430-20230703-00486 | Zhonghua Wei Zhong Bing Ji Jiu Yi Xue |
| Zhao 2020 Int Immunopharmacol | 31955067 |  | 10.1016/j.intimp.2019.106145 | International Immunopharmacology |
| Shen 2019 BMJ Open | 30782690 | PMC6352809 | 10.1136/bmjopen-2018-022896 | BMJ Open |
| Jiang 2023 Clin Exp Med | 36930382 |  | 10.1007/s10238-023-01029-w | Clinical and Experimental Medicine |
| Zhou 2024 Crit Rev Immunol | 38848289 |  | 10.1615/CritRevImmunol.2024051413 | Critical Reviews in Immunology |
| Li 2024 BMC Infect Dis | 39075364 | PMC11288105 | 10.1186/s12879-024-09516-5 | BMC Infectious Diseases |
| Song 2025 BMC Gastroenterol | 40751177 | PMC12315451 | 10.1186/s12876-025-04157-7 | BMC Gastroenterology |
| Akin Sen 2025 Medicine | 41189164 | PMC12537170 | 10.1097/MD.0000000000044979 | Medicine |

# Interpretation

All 10 included studies were identifiable in PubMed. This supports bibliographic verification of the included-study set but does not resolve the full PRISMA record count across all searched databases.
